# On Primary Syphilitic Buboes

**Published:** 1843-06

**Authors:** 


*On Primary Syphilitic Buboes.* By De Castlenau.—May a bubo of a truly syphilitic nature arise without antecedent chancre? Many writers on syphilis say *yes.* Many with one of the latest and ablest, Ricord, say *no.* De Castlenau has given the details of three cases, in which there seems to be no reason to doubt of the origin of the buboes. independently of antecedent external chancre. In one of these cases there was the very decisive evidence of the specific nature of the sore which followed the opening of the bubo, in the accidental inoculation of the surface in its neighborhood.

Pell (Marg.), aged 20, a week after connection, was seized with the ordinary symptoms of gonorrhœa, and by and by perceived a swelling in her right groin. The parts were examined with great care; no excoriation or ulceration was discovered either externally or internally; neither were there now any symptoms of gonorrhœa present. In the right groin there was a bubo, superficial, and on the point of giving way; in fact it did give way immediately after the visit. Next day another examination was made, but no chancre was discovered. The open bubo exhibited hard, inflamed, and sharply defined edges; the surface was gray and chancre-like, and suppurating copiously. Above the open sore a small pustule was observed. apparently the consequence of the natural inoculation of the pus poured out by the bubo. The wound was cauterized with nitrate of silver in substance. Two days later the sore had still the same chancrous character. The pustule was now a regularly rounded sore, with raised edges, sharply cut, and rather hard and red; its bottom was gray. A second pustule, like the first, was perceived on the skin to the inside of the principal sore. Three days afterwards this second pustule had become a chancrous sore, with the same characters as the other two. The patient, under appropriate treatment, recovered.--*Lond. and Edin. Med. Journ. Med. Sci.* Feb. 1843, from *Archiv. Gén. de Méd.,* Dec. 1842.

